# Effect of temperature and heart rate variability on Phantom T1 maps

**DOI:** 10.1186/1532-429X-17-S1-W24

**Published:** 2015-02-03

**Authors:** Vassilis Vassiliou, Ee Ling Heng, Evangelia Nyktari, Andreas Greiser, David Firmin, Dudley J Pennell, Peter Gatehouse, Sanjay K Prasad

**Affiliations:** 1 CMR, Royal Brompton Hospital, London, UK; 2 National Heart and Lung Institute, Imperial College London, London, UK; 3 Siemens, Germany, Germany

## Background

Phantoms are increasingly being used for quality assurance when undertaking T1 mapping for myocardial interstitial fibrosis to ensure long-term stability of methods applied in patients e.g. against scanner alterations, across multi scanners and multi centers. We sought to investigate whether temperature and heart rate (HR) variability could influence Phantom T1/ECV as such a variation will need to be considered in the quality assurance process.

## Methods

NiCl_2_-agarose gel phantoms [1] prepared in a standardised lab following a reproducible procedure as previously reported [2]. For the HR variability 4 phantoms were used to model native and post-contrast T1 of blood and myocardium; They were imaged with a prototype 11 HB MOLLI 5(3)3 (Siemens prototype WIP448B) at low resolution (HR>90bpm) and high resolution (<90bpm) for RR 1400-490ms representing HR 43-122 bpm (Avanto, Siemens, 1.5T) at steady temperature. For the temperature variability 4 phantoms were prepared to mimic short and long T1 at 25^o^C (Phantom B1=1915ms, B2=1040ms, B3=530ms, B4=280ms). A water bath was prepared where were the phantoms were immersed, starting from a temperature of 35 ^o^C cooling down to 15^o^C.

## Results

### Heart rate variability

**Figure 1:**
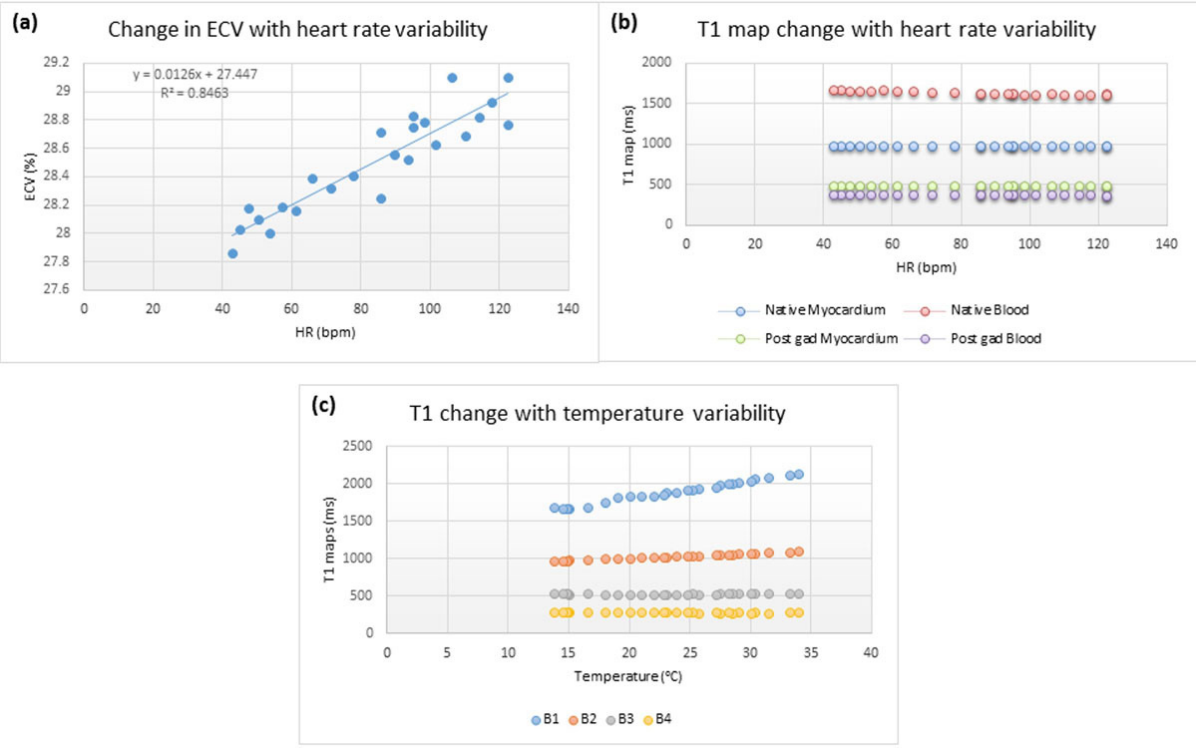
a) demosntrating a small but linear relationship between ECV and heart rate variability. b) shows that the native blood T1 are most affected. c) showing changes in T1 values with temperature variability. No ECV values were calculated as the T1 values did not mimic "human values".

There was a small but definite increase in ECV with increase in HR as shown in fig 1a. This followed a linear relationship (ECV= 0.013 x heart rate + 27.4, R2 = 0.85) suggesting an increase in ECV of 0.13% for every increase in 10bpm. For quality assurance it supports that HR should be indexed across vendors and sites. This difference is unlikely to be relevant for patients unless imaged in decompensated states (e.g. uncontrolled heart failure, fast atrial fibrillation). However, this change in ECV appears to be driven predominantly by a change in native blood T1 (Native blood T1= -0.78 X HR + 1700, R2 = 0.90) as shown in (fig 1b). Hence, in patients with extreme heart rates, it would appear that native myocardial T1 might be more accurate as this does not show variability with changes in HR (T1 native myocardium = 0.017 x HR + 981, R2 = 0.02).

### Temperature

With increase in temperature the T1 of Phantoms B1 and B2 appeared to increase (B1 T1= 23.86 x Temp +1323, R2 = 0.99 and B2 T1= 6.27 x Temp + 883, R2 = 0.98). The shorter T1 phantoms (B3 and B4) did not show an association with temperature changes, in accordance with previously published work from 1987 on Ni^2+^ doped phantoms [1].

## Conclusions

Heart rate variability has been shown to have a small but definite effect on the T1 values and ECV. Temperature variability can also affect the longer T1 values. Therefore, for successful T1 quality assurance with phantoms, both temperature and heart rate should be standardised. Such changes could further have implications for patients and new research is required to address this.

## Funding

NIHR Cardiovascular Biomedical Research Unit of Royal Brompton & Harefield NHS Foundation Trust and Imperial College London.
